# High-performance oxygen reduction electrocatalysis enabled by Ni_core_Pd_shell_ nanoparticles immobilized on MoS_2_ nanosheets

**DOI:** 10.1039/d5nr04406e

**Published:** 2026-03-05

**Authors:** Michail P. Minadakis, Yuta Sato, Ruben Canton-Vitoria, Kazu Suenaga, Nikos Tagmatarchis

**Affiliations:** a Theoretical and Physical Chemistry Institute, National Hellenic Research Foundation 48 Vassileos Constantinou Avenue Athens 11635 Greece tagmatar@eie.gr; b Research Institute of Core Technology for Materials Innovation, National Institute of Advanced Industrial Science and Technology (AIST) Central 5 1-1-1 Higashi Tsukuba Ibaraki 305-8565 Japan; c The Institute of Scientific and Industrial Research (SANKEN), The University of Osaka Mihogaoka 8-1 Ibaraki Osaka 567-0047 Japan; d Department of Materials Chemistry & Institute of Materials and Systems for Sustainability (IMaSS), Nagoya University Nagoya 464-8601 Japan; e Joining and Welding Research Institute, The University of Osaka Osaka 567-0047 Japan

## Abstract

The development of certain energy storage devices, such as fuel cells (FCs), as well as next-generation metal–air batteries (MABs), has increased during the first 25 years of the current century, mostly due to the startlingly rapid depletion rate of fossil fuels. The above devices operate electrochemically and commonly involve the oxygen reduction reaction (ORR). ORR mainly proceeds through a 4e^−^ pathway and suffers from both thermodynamic and kinetic limitations. Considering that the development of most next-generation electrocatalysts is still in its infancy, the use of noble metals is inevitable when the goal is to meet industry standards for commercially operating FCs in terms of the energy output while maintaining a low CO_2_ footprint. Palladium (Pd) appears to be the most economically viable and overall balanced choice in terms of cost/activity trade-off. However, the goal of utilizing low amounts of noble metals remains crucial for the development of electrocatalysts. The approach of constructing core–shell nanoparticles appears to be an attractive strategy for achieving atom economy and limiting the use of noble metals. The core–shell strategy relies on building a non-precious metal core and then displacing surface atoms with noble metal atoms to furnish a thin coating shell. The use of two-dimensional nanomaterials with suitable intrinsic properties and chemical/physicochemical tunability could also help to improve the steps towards sustainable electrocatalysts. Transition metal dichalcogenides (TMDs), especially MoS_2_, have not been explored as substrates for core–shell nanoparticles. This work focuses on immobilizing Ni_core_Pd_shell_ nanoparticles onto exfoliated semiconducting MoS_2_ nanosheets to furnish a novel ORR electrocatalyst. The Ni_core_Pd_shell_ nanoparticles were stabilized with 1-pyrenebutyric acid (PBA), and subsequently, were non-covalently immobilized on the 2H-MoS_2_ basal plane, to offer new ORR active sites along the pre-existing unsaturated Mo edges. The novel nanoensemble was fully characterized by spectroscopic, thermal and microscopic methods, to assess the interaction between the two moieties. Ultimately, the nanoensemble was studied as an alkaline ORR electrocatalyst through advanced electrochemical techniques, which unveiled the mechanism behind this interesting system, supporting its potential as a promising system for next-generation ORR electrocatalysts.

## Abstract 2

Interfacing exfoliated semiconducting MoS_2_ nanosheets with Ni_core_Pd_shell_ nanoparticles leads to a 4e^−^ alkaline oxygen reduction-active electrocatalyst. The nanoensemble exhibited superior to 30% Pd/C catalyst activity, with less than 1% Pd loading.

## Graphical abstract

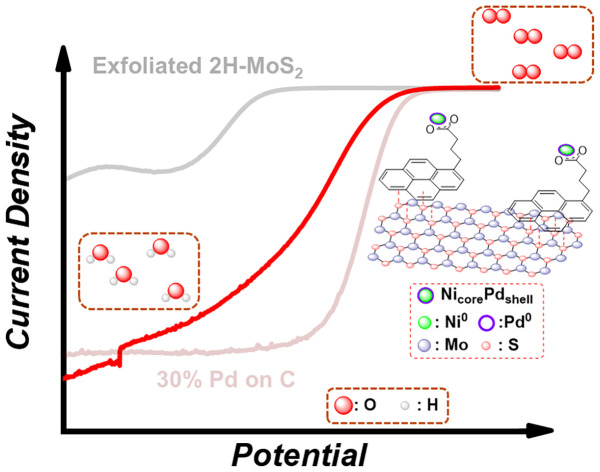

## Introduction

Currently, in the first part of the twenty-first century, society faces challenges related to meeting its growing energy demands. Given the alarmingly accelerated rate of fossil fuel depletion, industry has been preparing for a rapid transition to H_2_ production and to the use of electricity for vehicles and more sophisticated portable devices.^1^ In view of this, energy storage device development, namely of phosphoric acid fuel cells (PAFCs), proton exchange membrane fuel cells (PEMFCs), and direct methanol fuel cells (DMFCs), as well as new-generation, metal–air batteries (MABs), has increased during the past 25 years to keep pace with technological advances. The oxygen reduction reaction (ORR), which serves as the cathodic part of the electron process during their operation, is common to the above electrochemically operating devices. ORR is a key electrochemical process that is an integral part of a plethora of applications and proceeds *via* two distinct routes: the 2e^−^ and 4e^−^ pathways. The first produces H_2_O_2_ under either acidic (O_2_ + 2H^+^ + 2e^−^ → H_2_O_2_, *E*_eq_ = 0.695 V *vs.* RHE, pH = 0) or alkaline (O_2_ + H_2_O + 2e^−^ → HO_2_^−^ + OH^−^, *E*_eq_ = 0.693 V *vs.* RHE, pH = 13) conditions.^2^ This 2e^−^ pathway has emerged as the most promising process for industrial H_2_O_2_ production, and it is intended that it will substitute the standard Riedl–Pfleiderer (or anthraquinone) process.^3^ The other pathway leads to water as the final product under acidic (O_2_ + 4H^+^ + 4e^−^ → 2H_2_O, *E*_eq_ = 1.229 V *vs.* RHE, pH = 0) or alkaline (O_2_ + 2H_2_O + 4e^−^ → 4OH^−^, *E*_eq_ = 1.229 V *vs.* RHE, pH = 13) conditions.^2^ In most applications, the reaction proceeds *via* the 4e^−^ pathway, and it is impeded both thermodynamically—mainly due to sequential O–O bond cleavages (Δ*H*_O–O_ ∼ 500 kJ mol)—and kinetically, due to limitations arising from surface (adsorption–desorption) kinetics and low solubility-driven O_2_ transport.^4^ Since the ‘80s, the core for the design and construction of 4e^−^ ORR electrocatalysts has essentially been Pt embedded on a solid support, mostly high surface area carbon black (Vulcan®-type or Ketjenblack® carbon).^5^ Even though this electrocatalyst is characterized by high current densities that offer practicality for realistic applications, the usage ratio combined with the low abundance of Pt renders the overall cost drastically high. For this reason, during the next two decades, commercial applications transitioned to catalysts with lower noble metal contents; however, without compromising activity, such as Pt alloys with Co or Ni supported on carbon black, which remain the benchmark industry-level electrocatalysts to this day.^6^ The superiority of Pt resulted in increasing demand with limited supply, thus inevitably leading to a gradual cost increase (540$ per oz t††1 oz t = 1 troy ounce (∼31.1 grams) is the metric for noble metal mass. in 2000 to 970$ per oz t in 2025) that makes its use non-sustainable in the long run. To tackle this issue, from the early 2000s, nanostructure and facet engineering have been employed to increase the atom economy while retaining the activity.^7^ However, since the use of Pt was continued, this was only partly successful.

Certain new families of electrocatalysts, such as spinel-group oxides (NiCo_2_O_4_, MnCo_2_O_4_),^8^ MnO_*x*_/C, and in the early 2010s, perovskite oxides (La_0.8_Sr_0.2_Co_0.5_Mn_0.5_O_3_) and N,P-doped carbon nanomaterials, have emerged, expanding the use of alkaline conditions for FCs.^5^ Even though these materials are competitive against Pt, they are still in their infancy and subsequently cannot offer independence over Pt. In addition, noble-metal-free and metal-free ORR electrocatalysts require higher energy consumption to produce currents high enough to match those of a platinum group metal (PGM)-based catalyst.^9^ Based on this, it is clear that the use of noble metals is unavoidable when it comes to expecting an adequate energy output for commercially operating FCs while keeping CO_2_ footprint levels low. Since the cost of Ir is currently prohibitively high (415$ per oz t in 2000 to 4150$ per oz t in 2025) and Ru, while being more economical (200$ per oz t in 2000 to 630$ per oz t in 2025), is not sufficiently active for ORR, Pd seems to offer the best price/ounce *vs.* activity trade-off (690$ per oz t in 2000 to 800$ per oz t in 2025). Pd also has a higher natural abundance than Pt, roughly estimated to be almost 50 times higher. Therefore, it is reasonable to expect that the development of Pd-based catalysts will attract the interest of electrochemists worldwide.

A means of combining atom economy and low noble-metal is the preparation of core–shell nanoparticles. The core–shell strategy relies on building a non-precious metal core and then, by exploiting the different redox properties of each metal, displacing surface-located atoms with noble metal atoms to furnish a thin, noble metal-based coating shell.^10^ In this manner, the activity stemming from the noble metal is retained and is also amplified due to strain (changes in thermodynamics) and ligand (changes in kinetics) effects brought about by the non-noble core.^10,11^ Regarding the strain effect, lattice mismatches between core/shell metals induce tensile or compressive strain on surface noble-metal atoms, which strengthen or weaken the adsorbate binding affinity, respectively. Equivalently, the ligand effect is characterized by electron transfer between core/shell metal atoms that can directly affect the kinetics of the electrochemical reaction. Overall, this kind of synergy among different metal atoms can help minimize the precious metal content while simultaneously maximizing the efficiency, thus appearing as an attractive avenue to explore. Pd-based core–shell nanoparticles have been explored and applied in several instances, such as Suzuki–Miyaura coupling,^12,13^ electrochemical oxidative half-reactions (glucose oxidation and methanol oxidation and ethanol oxidation, and hydrogen oxidation),^14–17^ and mostly as ORR (both in acidic and alkaline environments) electrocatalysts.^15,17–19^

Since most metal-based catalysts use carbon black as the supporting material, another improvement step could be the switch from chemically inert supports to nanomaterials with intrinsic properties such as durability for mechanical and chemical stress, high conductivity that can provide more pathways for electron diffusion into the electrolyte, and tunability in terms of energy bandgap, chemical modification centers and overall dimensions. In that way, the nanomaterial would not only serve as the carrier but would also enable synergy with the metal nanoparticles to further improve their efficiency, help prevent particle aggregation, and enable uniform dispersion that would eventually aid the kinetics. While carbon nanomaterials have been well-explored,^15,19–22^ this field lacks current data on transition metal dichalcogenides (TMDs). Molybdenum disulfide (MoS_2_), a key representative of layered TMDs (MX_2_ type with M: Hf, Nb, Mo, W, Re, and X: S, Se, Te), is an interesting candidate for the development of advanced ORR electrocatalysts. This claim is supported by the well-studied abilities of MoS_2_: its conductivity that is equivalent to that of carbon nanomaterials, its superior pH resistance, its receptivity to interaction with interfacing elements, and the extensive knowledge of how to chemically modify it. It is critical to mention that to access the full potential of TMDs, exfoliation strategies are often utilized to engineer them into nanosheets, nanoflakes, *etc*., with an emphasis on liquid-phase exfoliation, as it is a facile, high-yield procedure.^23^

Herein, we focus on the non-covalent immobilization of Ni_core_Pd_shell_ nanoparticles onto exfoliated MoS_2_ nanosheets, thereby constructing a novel, ORR electroactive nanoensemble. While pristine MoS_2_ alone exhibits limited intrinsic activity toward ORR,^24^ its catalytic performance can be thermodynamically and kinetically enhanced through the presence of localized metal centers. In particular, the Mo atoms in MoS_2_ possess a favorable binding affinity for oxygen species, and their activity remains largely unaffected by delocalized oxidized sites within the lattice, as previously reported.^25,26^ To synthesize and stabilize the bimetallic core–shell nanoparticles, we employed 1-pyrenebutyric acid (PBA) as a non-covalent anchoring agent.^19^ While covalent functionalization typically yields more stable and well-defined hybrid systems, the non-covalent strategy presents several advantages: it enables a simpler and more versatile route to immobilize catalytically active species without significantly altering the intrinsic properties of either component, and it offers a cost-effective approach to assembling two functional moieties capable of cooperative behavior. Comprehensive characterization of the resulting Ni_core_Pd_shell_/MoS_2_ nanoensemble was conducted using a suite of spectroscopic techniques (UV-vis-NIR, PL, FT-IR, Raman and XPS), high-resolution transmission electron microscopy (HR-TEM) imaging, thermal analysis (TGA) and elemental mapping *via* energy-dispersive X-ray spectroscopy (EDS). These methods collectively confirmed the successful formation and structural integrity of the nanoensemble. Subsequently, the system was evaluated under alkaline conditions to assess its ORR performance and elucidate the synergistic interactions between the Ni_core_Pd_shell_ nanoparticles and the MoS_2_ support. These studies aimed to determine not only the catalytic contribution of each component but also the extent to which one enhances or modulates the activity of the other within the nanoensemble.

## Results and discussion

**Fig. 1:**
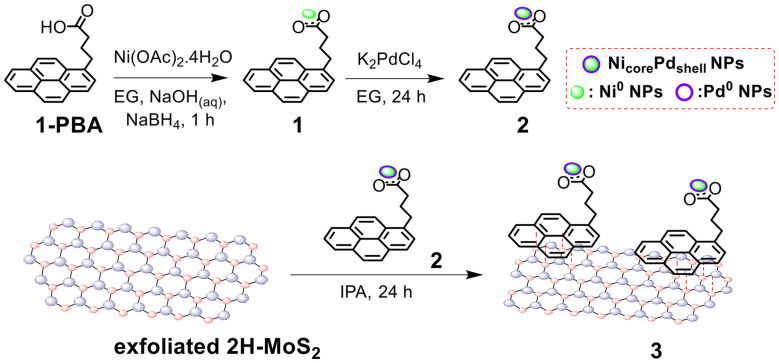
Synthetic path for Ni_core_Pd_shell_ nanoparticles and preparation route for Ni_core_Pd_shell_/MoS_2_ nanoensemble 3.

The preparation of the Ni_core_Pd_shell_ nanoparticles and subsequently the Ni_core_Pd_shell_/MoS_2_ nanoensemble is illustrated in Fig. 1. Ni_core_Pd_shell_ nanoparticles were prepared through a typical sequential synthesis commencing with the growth of Ni^0^ nanoparticles onto PBA in ethylene glycol (EG) using the strong reductant NaBH_4_. Then, interaction with an EG Pd^2+^ solution triggered the galvanic displacement process, where surface Ni atoms were exchanged with Pd atoms.^19,27^ The formation reaction is thermodynamically favored, as the standard potential for the redox couple (PdCl_4_^2−^)/Pd (+0.62 V *vs.* standard hydrogen potential, SHE) is higher than that of Ni^2+^/Ni (−0.257 V *vs.* SHE).^28^

**Fig. 2:**
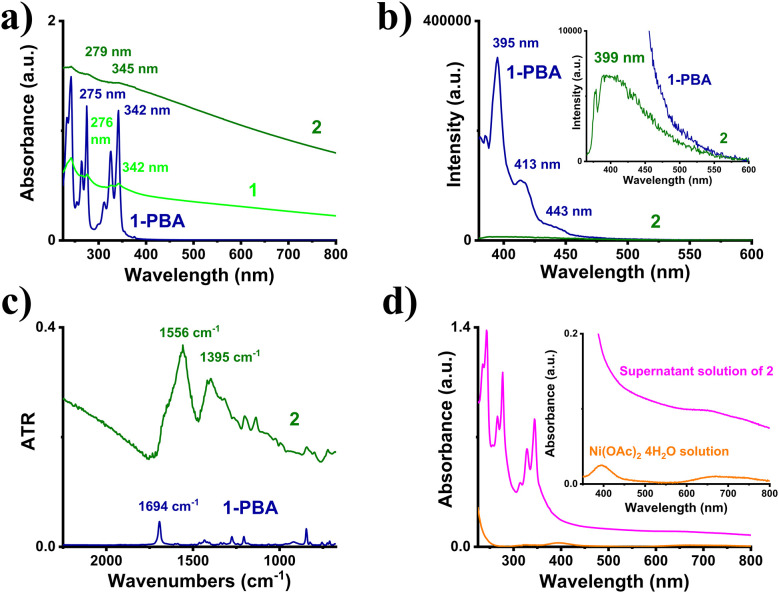
(a) UV-vis-NIR spectra in IPA, (b) PL spectra in IPA (inset: magnified image of Ni_core_Pd_shell_ fluorescence), (c) ATR spectra on a Ge crystal of PBA (navy), 1 (green), and 2 (olive), and (d) UV-vis-NIR spectra of the post-centrifugation, EG supernatant solution of 3 (magenta) and an EG Ni(OAc)_2_·4H_2_O (orange) solution (inset: magnified image of the UV-vis-spectra).

Nanoparticles in each step were grown onto and stabilized by the carboxyl groups of PBA molecules. The PBA-stabilized nanoparticles in each step were collected through controlled centrifugation and removal of nanoparticle-free supernatant solution. For the Pd shell, 30% coverage was targeted. Initially, by reacting Ni(OAc)_2_·4H_2_O with sodium borohydride in the presence of 1-PBA, PBA-stabilized Ni^0^ nanoparticles 1 were obtained quantitatively, following standard methodology based on previous reports.^19^ Then, by letting 1 interact with K_2_PdCl_4_ under ambient conditions for 24 h, the galvanic displacement process took place, eventually leading to the formation of PBA-stabilized Ni_core_Pd_shell_ nanoparticles 2. The above transformations were validated through drastic changes in UV-vis-NIR and photoluminescence (PL) spectra, as well as in FT-IR spectra. First and foremost, considerable alterations were observed in the 240–350 nm region of the UV-vis-NIR spectrum of 2. Specifically, as the Ni^0^ nanoparticles grew, the characteristic pyrene absorption peaks (strong signals at 242, 275, 320, and 342 nm) broadened, owing to the changes in the neighbouring electronic environment (Fig. 2a). Additionally, upon excitation at 340 nm to record the photoluminescence spectrum, the intense fluorescence of pyrene moieties was dramatically reduced by 98%, leaving only a faint peak at 399 nm, characteristic of quenched pyrene fluorescence (Fig. 2b). This was further supported by the FT-IR spectra recorded using the attenuated total reflectance (ATR) technique. The transition from PBA to PBA-stabilized nanoparticles was marked by the evident disappearance of the carboxylic carbonyl stretch mode located at 1694 cm^−1^ and the emergence of two intense, broad bands at 1556 cm^−1^ and 1395 cm^−1^, ascribed to the asymmetric and symmetric carboxylate group stretching vibrations, respectively (Fig. 2c). The broadening of the IR signals and the appearance of carboxylate group stretching vibrations corroborate the interaction with the nanoparticles. The % Pd load was also calculated by examining the post-reaction supernatant solution using UV-vis-NIR spectroscopy. The Ni^2+^ trace absorption band located at 620–760 nm (Fig. 2d), attributed to d–d transitions, was ultimately translated into a 27.9% Pd content per mg of 2. This percentage corresponds to a 93% reduction efficiency, and the final Ni : Pd ratio of the as-synthesized nanoparticles was estimated to be roughly 2.5 : 1.

The semiconducting MoS_2_ nanosheets were prepared through typical, chlorosulfonic acid-mediated liquid-phase exfoliation.^29^ The goal was to produce nanosheets in a high yield that retain their semiconducting character and are low in defects. The nature of the exfoliated 2H-MoS_2_ nanosheets was corroborated by spectroscopic methods. As seen in Fig. S1a, the features in the UV-vis-NIR spectrum of exfoliated 2H-MoS_2_ were more pronounced compared to the bulk material, pointing to the existence of a few-layered material. The hallmarks of semiconducting MoS_2_ are the direct excitonic transitions A and B, located at 681 and 622 nm, respectively, that originate from energy splitting of the valence band and spin–orbit coupling.^30–32^ Raman spectroscopy (Fig. S1b) also supports the nature of the exfoliated material, as an increase in the intensity of the longitudinal acoustic phonon mode 2LA(M), which reflects the number of lattice defects (S vacant sites), is observed as expected, as all exfoliation processes induce defects in the final nanomaterial. Furthermore, the widening of the distance between the E^1^_2g_ and A_1g_ modes by 2–3 cm^−1^ is characteristic of the exfoliated nature.^30,33^

**Fig. 3:**
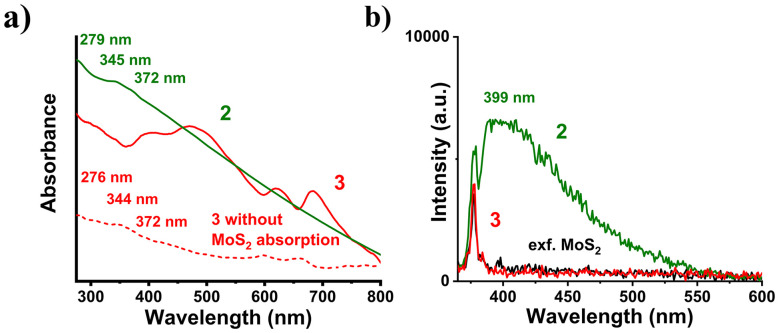
(a) UV-vis-NIR spectra in IPA, and (b) PL spectra (*λ*_exc_ = 340 nm) in IPA of 2H-MoS_2_ (black), 2 (olive) and 3 (red); the red dotted line shows the solution of 3 with the absorbance of the MoS_2_ solution subtracted.

The Ni_core_Pd_shell_/MoS_2_ nanoensemble 3 was prepared by simply mixing 2 and exfoliating 2H-MoS_2_ nanosheets in isopropanol (IPA), under ambient conditions and an inert N_2_ atmosphere. After 24 hours, the mixture was mildly centrifuged (6000 rpm) to remove unbound and weakly bound PBA-stabilized Ni_core_Pd_shell_ nanoparticles (*vide supra*, Fig. 1). The interaction between the two species was again validated through UV-vis-NIR spectroscopy. As seen in Fig. 3a, when the spectrum of 3 is compared to that of 2, no features other than those of 2H-MoS_2_ are observed, masking those related to Ni_core_Pd_shell_ absorption. As 3 is a nanoensemble, the absorbances of both species are superimposed. Therefore, experimental subtraction of MoS_2_ absorbance should reveal characteristics of 2 in the final spectrum. Indeed, this appears to be the case as seen in Fig. 3a, where the 276 nm, 344 nm, and 372 nm signals that emerge can be clearly ascribed to nanoparticle absorbance, supporting the initial hypothesis. Further evidence comes from PL assays of 3, where the interaction of components is studied in the excited state. It was observed that the fluorescence arising from PBA-stabilized nanoparticles is completely quenched (Fig. 3b). This is attributed to MoS_2_ nanosheets acting as electron acceptors,^34^ therefore reabsorbing the photons produced by 2 and de-exciting non-radiatively. Additional support comes from the ATR-IR spectra, where in the spectrum of 3, the two new vibrational modes located at 1654 cm^−1^ and 1378 cm^−1^ can be ascribed to the vibrational modes of the PBA chain carboxylate groups of 2, further confirming the interaction between the above two species (Fig. S2). Raman spectroscopy revealed that no changes occurred on the basal plane or edges of the 2H-MoS_2_ surface, as the spectra with and without 2 exhibit no differences (Fig. S3a). This was also supported by statistical Raman spectral maps on 30 × 30 μm areas, where the point of interest is the intensity ratio A_1g_/2LA(M). Practically, no difference was observed between 2H-MoS_2_ and 3, further supporting the above conclusion (Fig. S3b and c).

**Fig. 4:**
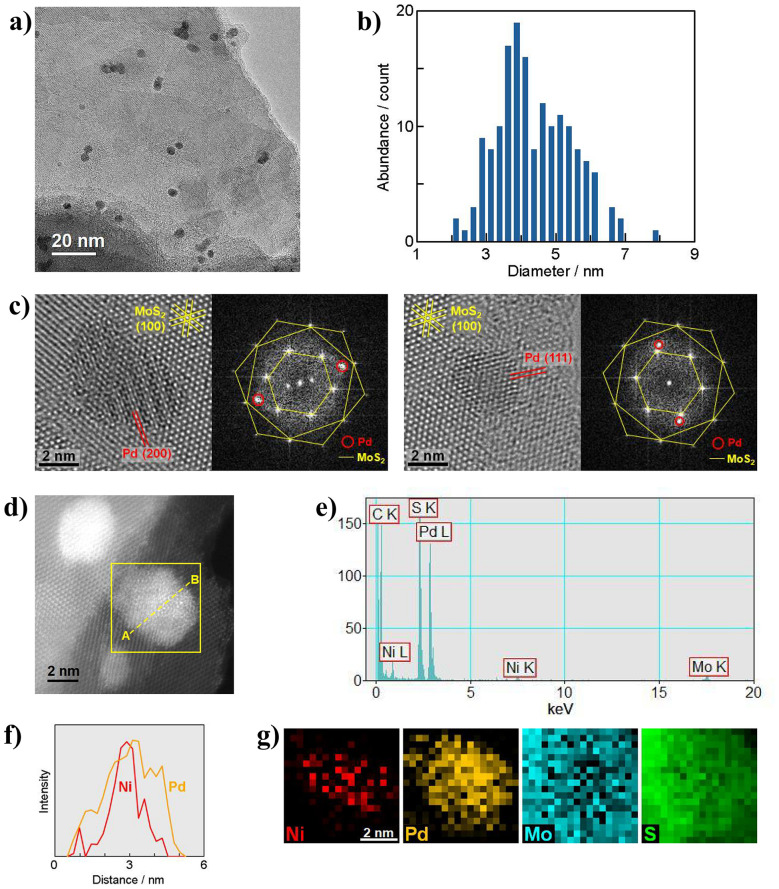
(a) TEM image of 3. (b) Size distribution of the Ni_core_Pd_shell_ nanoparticles. (c) HRTEM images of 3 and their FFT images. (d) Annular dark-field STEM image of 3. (e) EDS spectrum for the area surrounded by a yellow square in (d). (f) Intensity profiles of Ni and Pd from EDS line scanning along the dashed line A–B in (d). (g) EELS elemental maps of Ni, Pd, Mo and S for the area surrounded by a yellow square in (d).

Visualization of 3 was performed through transmission electron microscopy (TEM) and scanning TEM (STEM) combined with energy-dispersive X-ray spectroscopy (EDS) and electron energy loss spectroscopy (EELS). A low magnification TEM image in Fig. 4a proves that Ni_core_Pd_shell_ nanoparticles are finely dispersed onto MoS_2_, forming nanoensembles. The size distribution of the Ni_core_Pd_shell_ nanoparticles based on their TEM and STEM images is shown in Fig. 4b. The average nanoparticle size and the standard deviation were determined to be 4.39 nm and 1.08 nm, respectively. In the high-resolution (HR) TEM images of typical nanoparticles (Fig. 4c), the lattice fringes of face-centred cubic (fcc) Pd metal, such as (200) and (111), are identified by the interplanar spacings of 0.194 nm and 0.225 nm, respectively. Additionally, the fast Fourier transform (FFT) showcases high crystallinity, displaying the characteristic MoS_2_ lattices together with weak yet measurable Pd contributions. Although determination of Ni core structures in detail needs further analysis, the Ni–Pd core–shell configuration was confirmed by means of STEM-EDS/EELS, as shown in Fig. 4d–g. The EDS line scan profiles (Fig. 4f) and the EELS elemental maps (Fig. 4g) clearly show that the distribution of Ni is localized at the centre of the nanoparticle and surrounded by Pd.

**Fig. 5:**
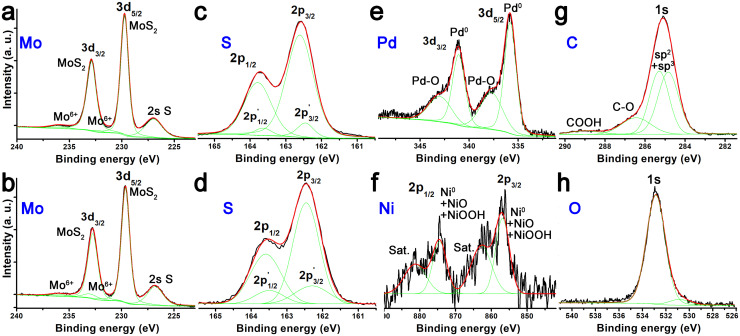
XP spectra of Mo and S for (a and c) exfoliated 2H-MoS_2_, and Mo, S, Pd, Ni, C and O (b and d–h) for nanoensemble 3.

XPS provides detailed insight into the electronic states of the elements that constitute nanoensemble 3. As shown in Fig. 5, the Mo 3d_5/2_ and 3d_3/2_ doublet at 229.72 and 232.87 eV, characteristic of exfoliated 2H-MoS_2_, remains intact in nanoensemble 3, with only negligible oxidation features observed at 232.39 and 236.20 eV.^35^ Similarly, the S 2p_3/2_ and 2p_1/2_ signals associated with the basal plane of MoS_2_ at 162.61 and 163.79 eV, as well as those related to edge sites at 162.44 and 163.63 eV, are also preserved in nanoensemble 3 with only minor variations.^36^ These results indicate that the MoS_2_ nanosheets remain chemically intact during the PBA-supported Ni_core_Pd_shell_ nanoparticle immobilization process. Focusing on nanoensemble 3, Pd was clearly detected, exhibiting predominant Pd 3d_5/2_ and 3d_3/2_ peaks at 334.82 and 341.07 eV, corresponding to metallic Pd, together with shoulders at 337.75 and 343.07 eV associated with Pd–O species.^37^ While metallic Pd is likely to be located in the inner regions of the nanoparticles, the Pd–O component can be attributed to surface-exposed Pd atoms. These oxide species may enhance the wettability of the material and could also play a role in the electrochemical oxygen reduction reaction discussed later. The Ni 2p region was very noisy due to extensive coverage by Pd, which shields the Ni signal and complicates its interpretation. Nevertheless, broad features centered at 856.83 and 874.42 eV (Ni 2p_3/2_ and 2p_1/2_, respectively) suggest a mixture of metallic Ni and oxidized species such as NiO and NiOO-R.^38,39^ The presence of these oxide components can be related to covalent linkages between Ni and oxygen-containing moieties from the PBA unit. Regarding the C 1s region, nanoensemble 3 exhibits peaks at 284.84 and 285.31 eV, corresponding to sp^2^ and sp^3^ carbon, respectively. In addition, a weak contribution from carboxylic acid groups is observed at 289.21 eV, while a strong signal at 286.5 eV indicates that the PBA was largely reduced by NaBH_4_ and consumed during nanoparticle stabilization, in agreement with the IR results. The O 1s region could not be reliably analyzed because it is masked by SiO_*x*_ species from the Si substrate. Finally, upon approaching the XPS detection limit, the Ni content relative to Mo was estimated to be ∼1%, in agreement with other techniques.

The thermal profile of 3 was also analyzed using TGA, and letting the sample decompose under an inert N_2_ atmosphere (Fig. S4). With 2H-MoS_2_ as a reference substrate, 3 exhibited an almost 4% weight loss in the temperature range 250–450 °C, where the two decomposition profiles showed differences, attributed to the organic PBA chains, ultimately translating into a 0.28% Pd content per mg of 3. Overall, Ni_core_Pd_shell_/MoS_2_ nanoensemble 3 has a 100-fold lower Pd content compared to the state-of-the-art, commercially available 30% Pd/C electrocatalyst.

**Fig. 6:**
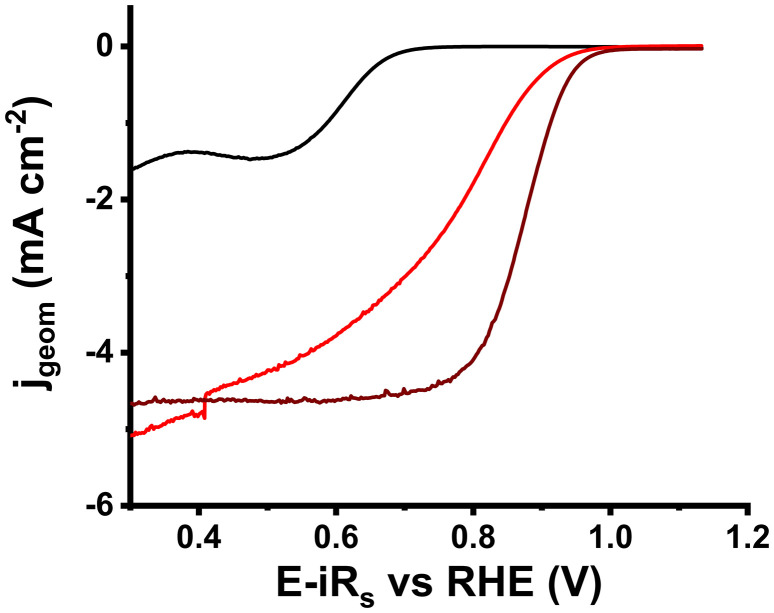
*iR*-Compensated, N_2_-background corrected LSV curves of 2H-MoS_2_ (black), 3 (red), and 30% Pd/C (wine).

Nanoensemble 3 was examined for alkaline ORR electrocatalysis in an O_2_-purged, aqueous 0.1 M KOH electrolyte. To unveil the characteristics of the potential electrocatalyst, linear sweep voltammograms (LSVs) were recorded using a rotating ring-disk electrode (RRDE) with glassy carbon (GC) as the conductive material, rotation speed of 1600 rpm and scan rate 5 mV s^−1^. For comparison, the catalytic activity of the 30% Pd/C reference nanomaterial was also estimated under the same conditions. To obtain a representative picture, LSVs were first recorded in an N_2_-saturated electrolyte and subtracted from those in O_2_. All LSVs are presented with 100% compensation for internal electrolyte resistance (*R*_s_). All values are presented *versus* the reversible hydrogen electrode (RHE) for clarity in terms of comparison with other works in the literature. As seen in Fig. 6, regarding the start of the reaction, the onset potential (*E*_ons_) of 3 is roughly located at 0.924 V, less than 40 mV further on from that of the 30% Pd/C (0.961 V). This underscores the impact of Pd on MoS_2_ as the latter exhibits *E*_ons_ at 0.675 V, clearly further from that of the state-of-the-art material. Another metric that is complementary to *E*_ons_ is the half-wave potential (*E*_1/2_), namely the voltage value at half of the maximum current density. *E*_1/2_ for 3 was found to be 0.747 V, 125 mV lower than that of 30% Pd/C (0.872 V). Even though these two values tend to behave equivalently for a certain material, the much lower *E*_1/2_ for 3 could be ascribed to the poorly formed plateau region. In terms of the current yield, the diffusion-limited current density value (*j*_d_) of 3 was found to be 5.06 mA cm^−2^, meaning that the Ni_core_Pd_shell_ nanoparticles interfacing with MoS_2_ nanosheets grant the nanoensemble the ability to allow increased current flow by almost 0.4 mA cm^−2^, compared to 30% Pd/C (*j*_d_ = 4.67 mA cm^−2^). A valuable index for the ORR electroactivity of a material is the mass-transport-corrected (kinetic) current density (*j*_k_) at 0.9 V *vs.* RHE, as industry standards dictate.^40,41^ In our case, due to the uncommon shape of the LSV curve of 3, *j*_k_ was calculated at 0.85 V *vs.* RHE, where kinetically controlled and mixed (both kinetic and diffusion control) regions meet. The kinetic current density for nanoensemble 3 was found to be reduced by 37% (1.19 mA cm^−2^) compared to that of 30% Pd/C (1.91 mA cm^−2^).

**Fig. 7:**
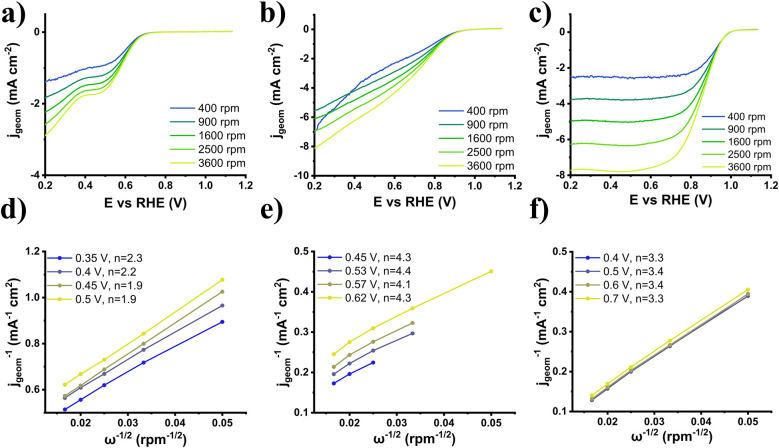
(a–c) LSV curves for different rotation speeds, and (d–f) K–L plots for 2H-MoS_2_ (a and d), 3 (b and e), and 30% Pd/C (c and f).

Considering the overall electrocatalytic behavior of nanoensemble 3, it can be safely placed among the 4e^−^ path ORR electrocatalysts, mechanism-wise. This marks the most important impact of Pd on the nanoensemble, the transition from the 2e^−^ characteristics of MoS_2_ to a 4e^−^ system. To corroborate this attribute, Koutecký–Levich (K–L) analysis is required. LSVs with various rotation speeds, 400–3600 rpm in our case, were recorded, and current density values for different voltages at the diffusion-controlled/plateau region were determined. Subsequently, the inverse of the geometrical surface corrected current density (*j*_geom_^−1^) was plotted against the inverse of the square root of the rotation speed (*ω*^−1/2^). It is expected that the derived points present good linearity, from the slope of which the electron transfer number (*n*) is derived. This method allows for entanglement between mass transport limitations and intrinsic electrochemical kinetics, and thus the ability to focus on the reaction mechanism. The observed recorded current increase with increased rotation speed is ascribed to the reduction of the diffusion layer at a higher speed. The series of LSVs is illustrated in Fig. 7. The number of electrons (*n*_KL_) for 3 was found to be around 4.1–4.4 in comparison with semiconducting MoS_2_ values of 1.9–2.3, while for 30% Pd/C, the number was estimated to be around 3.3–3.4. The clear transition from a selective 2e^−^ path to a selective 4e^−^ path concerning nanoensemble 3 is once again validated, as well as the superiority over the dominant 4e^−^ path of 30% Pd/C.

To obtain the complete picture of the ORR pathway selectivity, the electron transfer number was also calculated through the RRDE approach (*n*_RRDE_) and the current recorded from the Pt ring, along with an estimation of the H_2_O_2_ percentage.^42^ In the range 0.2–0.6 V *vs.* RHE, *n*_RRDE_ for exfoliated MoS_2_ was calculated as ∼1.9, while for 3 and Pd/C it was calculated as ∼3.4 and ∼3.3, respectively, with % H_2_O_2_ production yields of 90%, 35% and 37%, respectively (Fig. S5). The above data confirm both the change in behavior of nanoensemble 3 and the selectivity for the 4e^−^ ORR pathway.

**Fig. 8:**
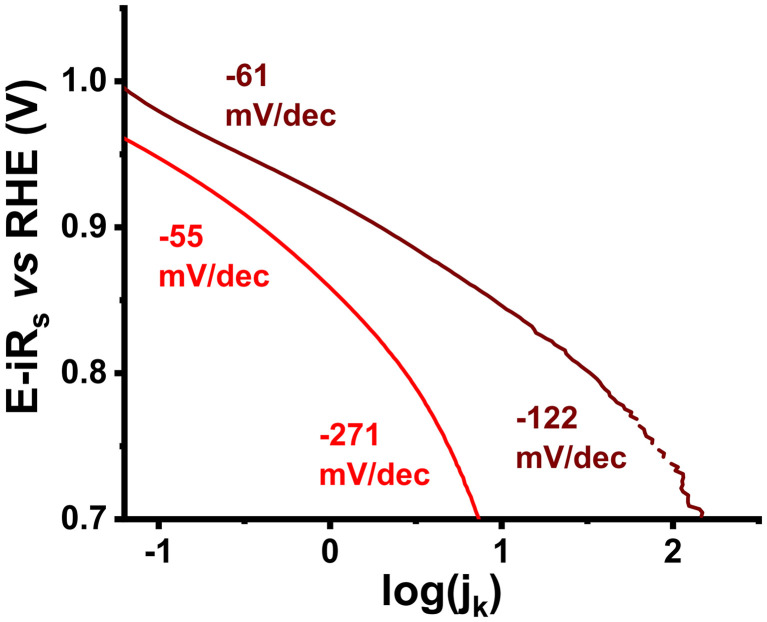
Tafel slopes of 3 (red), and 30% Pd/C (wine).

In order to delve into the ORR mechanism for 3, Tafel slopes were extrapolated from LSV curves (Fig. 8). Typically, ORR Tafel slopes consist of two regions, namely, the low current density region (I) and the high current density region (II). Based on the K–L fittings, good linearity for both 3 and Pd/C entails first-order kinetics. The slope for 3 in region I was calculated to be 55 mV dec^−1^, while that for 30% Pd/C was 61 mV dec^−1^. Regarding region II, the Tafel slope for 3 was found to be 271 mV dec^−1^, more than double that of 30% Pd/C (122 mV dec^−1^). At the low current region, nanoensemble 3 appears to behave similarly to 30% Pd/C, as Pd sites play the most important role and the reaction kinetics are purely governed by electron transfer. In more detail, the conditions at low current densities can be described by the Temkin model, where the increasing surface oxide coverage gradually accelerates the reaction, as 3 is a heterogeneous system in terms of dispersion of Ni_core_Pd_shell_ nanoparticle active centers. At higher currents, Pd site saturation occurs, the mechanistic approach switches to the Langmuir model, and in the case of 3, due to Pd sites not being uniformly distributed on the MoS_2_ plane, the reaction slows down as the mass transport contribution becomes important. The higher value for the Tafel slope is attributed to a mixture of kinetic and non-kinetic contributions (mass transport, ohmic drops, *etc*.) affecting the reaction's progress, eventually causing its deceleration.

**Fig. 9:**
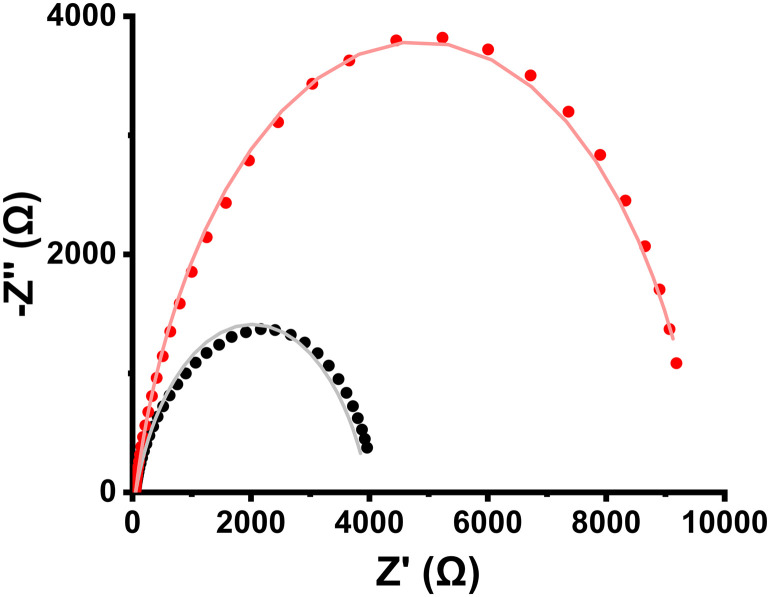
Nyquist plots (dots) and fittings (lines) of 2H-MoS_2_ (black), 3 (red), and 30% Pd/C (wine ).

Another aspect of electrochemical kinetics is the determination of the charge transfer resistance (*R*_ct_), that is, the resistance an electron experiences when crossing the electrochemical double layer between the electrode surface and the electrolyte. We gain insight into this metric through the electrochemical impedance spectroscopy (EIS) technique. As shown in Fig. 9, nanoensemble 3 exhibited twice as high *R*_ct_ (9.55 kΩ) as either the starting material (4.21 kΩ) or the reference 30% Pd/C (4.19 kΩ). All the materials can be described by a typical basic Randles circuit because they behave as non-ideal capacitors (Fig. S6). Although the MoS_2_ surface is decorated with ORR active centers, the absence of conjugation in organic PBA chains impedes the flow of electrons from the Ni_core_Pd_shell_ nanoparticles to the surface of the pseudoelectrode. Such a case is linker-dependent and can be mitigated with the use of highly conjugated organic spacers.

In industry metrics, apart from *E*_ons_ and *j*_d_, important aspects of an ORR electrocatalyst are the mass (MA) and specific (SA) activities. The MA demonstrates the catalytic capability of a catalyst per unit of noble metal mass, while the SA reflects the intrinsic catalytic activity of the noble metal catalyst per unit of exposed area.^43^ To get access to these elements, the electrochemically active surface area (ECSA) needs to be determined. In the case of noble metal-based electrocatalysts (mostly Pt and Pd) and oxygen catalysis, the ECSA can be derived from cyclic voltammetry (CV) by locating the oxide reduction peak (PdO in our case), calculating the charge of the oxide reduction peak (*Q*_o_), comparing it with the charge acquired by the adsorption of an oxygen monolayer on the Pd surface per unit of area (*Q*^ref^_o_), and normalizing it by the Pd loading of the pseudoelectrode. We assumed a PdO *Q*^ref^_o_ of 424 μC cm^−2^ for the calculations outlined in the SI (Fig. S7).^44^ The ECSA value for nanoensemble 3 was estimated to be 530 cm^2^ mg_Pd_^−1^, slightly higher than that for 30% Pd/C (450 cm^2^ mg_Pd_^−1^), yet with an almost 75% lower Pd loading. Conversion of the ECSA to the MA was carried out by using *j*_k_ values at the corresponding potential. The MA for nanoensemble 3 was calculated and found to be 84 mA mg_Pd_^−1^, more than double that of 30% Pd/C (37 mA mg_Pd_^−1^). In the work of Jiang *et al.*, which presents a case similar to our Ni_core_@Pd_shell_ nanoparticles, half the MA of nanoensemble 3 was reported (42 mA mg_Pd_^−1^) under similar conditions.^18^ In a past work of our research team, for an equivalent system but with graphene sheets as the substrate, a MA of ∼520 mA mg_Pd_^−1^ was estimated, but this corresponded to a Pd content of 5.6%, where again the % Pd content-activity trade-off was inferior to the one reported in this work.^19^ The superiority of 3 compared to the reference material is attributed to the combined effects of the increased electroactivity of Pd atoms due to internal Ni_core_Pd_shell_ nanoparticle-occurring phenomena and the MoS_2_ nanosheets that offer ample anchoring sites for the stabilized Ni_core_Pd_shell_ nanoparticles, thus preventing aggregation due to π–π stacking. In view of the above, it can be understood that the exposed active surface area is greater in the nanoensemble. This is corroborated by the calculation of the SA, which was found to be 0.158 mA cm^−2^, almost double the value for 30% Pd/C (0.082 mA cm^−2^). All the above activity elements that have been calculated are summarized in Table 1.

**Table 1:** Summary of electrochemical data for nanoensemble 3 and 30% Pd/C

Material	*j*_d_ (mA cm^−2^)	*E*_on_ (V)	*E*_1/2_ (V)	*j*_k_ (mA cm^−2^)	Electron transfer number (*n*_KL_)	Tafel slope (mV dec^−1^)	ECSA (cm^2^ mg_Pd_^−1^)	MA (mA mg_Pd_^−1^)	SA (mA cm^−2^)
3	5.06	0.924	0.747	1.19@0.85 V	4.1–4.4	−55/−271	530	84	0.158
30% Pd/C	4.67	0.961	0.872	1.91@0.9 V	3.3–3.4	−61/−122	450	37	0.082

**Fig. 10:**
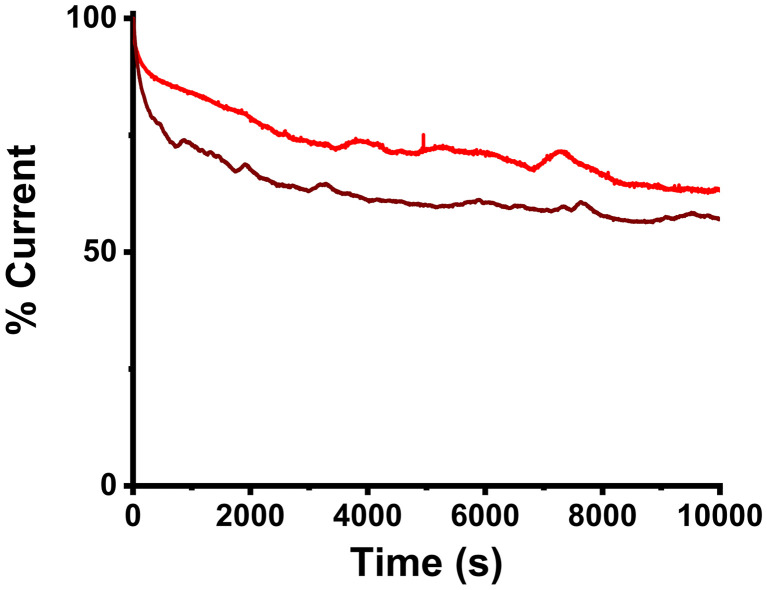
Chronoamperometric curves of 3 (red) and 30% Pd/C (wine) after 10 000 s at operating voltages of 0.382 V and 0.432 V, respectively.

Finally, to study the long-term performance of 3, chronoamperometry (CA) was employed, where the produced current at a set potential for each material under investigation was recorded. As seen in Fig. 10, after three hours of continuous operation, 3 loses approximately 37% of the initial current output, whereas 30% Pd/C has its current yield reduced by almost 43%. It appears that nanoensemble 3 behaves equivalently to the reference material, yet it slightly reduces the current loss.

## Conclusions

In summary, we prepared a novel, semiconducting MoS_2_-based nanoensemble that hosts PBA-stabilized Ni_core_Pd_shell_ nanoparticles. These nanoparticles induce a significant enhancement on the ORR electrocatalytic performance enabling a mechanistic switch from the original 2e^−^ pathway of MoS_2_ to a more efficient 4e^−^ reduction process. This switch is critical in improving the selectivity and efficiency of the ORR, a key reaction in fuel cells and metal–air batteries. When benchmarked against the state-of-the-art commercial Pd/C electrocatalyst, the newly synthesized nanoensemble demonstrates an equivalent onset potential coupled with a notably higher diffusion-limited current density. This indicates superior catalytic kinetics and enhanced electron transport. Remarkably, the nanoensemble achieves high selectivity towards the 4e^−^ ORR pathway, while maintaining substantially higher mass and specific activities. This performance is achieved with a palladium content of only 0.28%, which is over 100 times lower than the 30% Pd loading in the conventional Pd/C catalysts, representing a substantial reduction in precious metal usage. Furthermore, the Ni_core_Pd_shell_/MoS_2_ nanoensemble exhibits improved durability under chronoamperometric conditions, by retaining 6% more current over prolonged operation. This performance underscores its potential for practical applications where long-term operational stability is critical. This work highlights the profound impact of rationally designed nanoscale modifications in tailoring electrocatalytic properties. It exemplifies how the strategic incorporation of cost-effective building blocks into nanocatalysts can yield a performance that rivals or surpasses that of conventional state-of-the-art materials, offering a promising avenue for sustainable and economically viable ORR electrocatalysts.

## Author contributions

Minadakis M. P.: methodology, validation, formal analysis, investigation, data curation, writing – original draft, writing – review & editing, visualization; Sato Y.: methodology, validation, formal analysis, investigation, resources, data curation, writing – original draft, writing – review & editing; Canton-Vitoria R.: methodology, validation, formal analysis, investigation, data curation, writing – review & editing; Suenaga K.: methodology, validation, formal analysis, investigation, resources, data curation, writing – original draft, writing – review & editing; Tagmatarchis N.: conceptualization, resources, writing – review & editing, supervision, project administration, funding acquisition.

## Conflicts of interest

There are no conflicts to declare.

## Supplementary Material

NR-018-D5NR04406E-s001

## Data Availability

The data supporting this article have been included as part of the supplementary information (SI). Supplementary information is available. The SI contains the following: Materials, methods, and instrumentation used; Mathematical equations used for the calculations; Detailed experimental procedures; Supplementary Figures. See DOI: https://doi.org/10.1039/d5nr04406e.
