# Supplementary material for: High-performance oxygen reduction electrocatalysis enabled by NicorePdshell nanoparticles immobilized on MoS2 nanosheets
Source: Nanoscale. 2026 Mar 5;18(15):8329–38. doi: 10.1039/d5nr04406e (PMC12998532; doi:10.1039/d5nr04406e)
Supplement: NR-018-D5NR04406E-s001 [file NR-018-D5NR04406E-s001.pdf]

Supplementary Information (SI) for Nanoscale.
This journal is © The Royal Society of Chemistry 2026

ELECTRONIC SUPPLEMENTARY INFORMATION

High-performance oxygen reduction electrocatalysis enabled by $Ni_{core}Pd_{shell}$ nanoparticles immobilized on MoS_2 nanosheets

Michail P. Minadakis,[a] Yuta Sato,[b,c] Ruben Canton-Vitoria,[d,e] Kazu Suenaga,[c] and Nikos Tagmatarchis*[a]

[a] *Theoretical and Physical Chemistry Institute, National Hellenic Research Foundation, 48 Vassileos Constantinou Avenue, Athens 11635, Greece. Email: tagmatar@eie.gr*

[b] *Research Institute of Core Technology for Materials Innovation, National Institute of Advanced Industrial Science and Technology (AIST), Central 5, 1-1-1 Higashi, Tsukuba, Ibaraki 305-8565, Japan.*

[c] *The Institute of Scientific and Industrial Research (SANKEN), The University of Osaka, Mihogaoka 8-1, Ibaraki, Osaka 567-0047, Japan.*

[d] *Department of Materials Chemistry & Institute of Materials and Systems for Sustainability (IMaSS), Nagoya University, Nagoya 464-8601, Japan.*

[e] *Joining and Welding Research Institute, The University of Osaka, Osaka 567-0047, Japan.*

* *Corresponding author*

Materials and methods

All chemicals and solvents were commercially available and used as received unless otherwise stated. All solvents as well as chlorosulfonic acid (HSO_3Cl) were purchased from Honeywell (Diegem, Belgium) and were of analytical grade. Molybdenum disulfide powder (particle size ~6 μm) was purchased from Sigma Aldrich (Merck KGaA group, Darmstadt, Germany). $Ni(OAc)_2\cdot 6H_2O$, $NaBH_4$, and K_2PdCl_4 were purchased from commercial sources and used as received. PTFE filters (47 mm diameter, 0.2 μm pore size) were purchased from ADVANTEC, Japan.

Instrumentation

Probe sonication was performed with a Bandelin Sonopuls Ultrasonic Homogenizer HD 3200 equipped with a flat head probe (VS-70T), running at 35% (87.5 W) of the maximum power (250 W).

Mid-infrared spectra in 675-4000 cm^{-1} region were acquired on a Fourier transform IR spectrometer (Tensor II, Bruker Optics) equipped with a single reflection Ge ATR accessory (Miracle by PIKE Technologies). Typically, 100 scans were acquired at 4 cm^{-1} resolution.

Micro-Raman scattering measurements were performed at room temperature (25 ± 2 °C) in the backscattering geometry using a RENISHAW inVia Raman spectrometer equipped with a CCD camera and a Leica microscope. A 1200 lines mm^{-1} grating was used for all measurements, providing a spectral resolution of ± 1 cm^{-1}. As the excitation source, a He/Ne laser line of 633 nm was used. Acquisitions were collected at the 100-2000 cm^{-1} region on extended mode, with 10 s of exposure time and laser power 50% (corresponding to ~0.3 mW cm^{-2}) to avoid overheating of the samples. The laser spot was focused on the sample surface using a long working distance 50x objective lens. Raman spectra were collected after map image acquisition was conducted on different areas of the sample and recorded with Peltier cooled CCD camera. The intensity ratio A_{1g}/2LA(M) was obtained by dividing the peak intensities, following any baseline corrections. For the Raman spectral maps, 3 different areas of 121 acquisition points were scanned for every sample, and the most representative as well as closest to the average of sole acquisitions is outlined. The data were processed with Renishaw Wire V4.3 and Origin Pro 8 software.

UV-vis-NIR absorption spectra were recorded on a PerkinElmer (Lambda 19) UV-Vis-NIR spectrophotometer (200-2500 nm) with PMT detector (deuterium lamp as the light source for UV, halogen lamp for Vis/NIR), at a scan speed of 480 nm min^{-1}, 2 nm slit width and 1 nm interval for data acquisition.

Steady-state photoluminescence spectra were recorded on a Fluorolog-3 Jobin Yvon-Spex spectrofluorometer (model GL3-21). The samples were prepared in IPA (Honeywell, >99.9% purity), irradiated at λ_{exc} = 340 nm and fluorescence was detected at the 370-700 nm range with

excitation and emission slits adjusted to 2 nm, integration time at 0.1 s and data input every 0.2 s.

Transmission electron microscopy (TEM) and scanning TEM (STEM) were performed using a JEOL JEM-2100F-based electron microscope equipped with double JEOL Delta spherical aberration correctors at an electron accelerating voltage of 60 kV. TEM images were recorded by a Gatan Ultrascan charge-coupled device (CCD) camera. Energy-dispersive X-ray spectroscopy (EDS) and electron energy loss spectroscopy (EELS) were carried out using double JEOL Centurio detectors and a Gatan Quantum electron spectrometer, respectively.

Thermogravimetric analysis (TGA) was performed on a lab-scale Q50 instrument V20.13 Build 39 (TA, Eschborn, Germany) under inert N2.0 (99.9%) purity grade N_2 atmosphere and standard balance (40 mL min^{-1}) and sample (60 mL min^{-1}) gas flows. In a typical experiment, 1-2 mg of the nanomaterial under analysis were placed in a Pt pan (prior to the experiment, any impurities on the Pt pan were burned off using a commercial blowtorch). Subsequently, the material was subjected to the following experimental procedure: temperature equilibration at 100 °C, isotherm at 100 °C for 10 min., and ramp up to 800 °C at a 10 °C min^{-1} rate. Mass changes were recorded as a function of temperature. Differential thermograph (DTG) was also recorded to aid with the determination of the decomposition profile of each compound. The results were analyzed with Instrumental analysis V5.5.23 and Origin Pro 8 software.

X-ray photoelectron spectroscopy (XPS) was performed by forming an ultrathin film of the material on the surface of water by carefully contacting a few drops of a 1 mg/mL dispersion of the material in MeOH. The film was subsequently collected using a N-doped silicon wafer. The films were dried for 15 min at 100 °C in air. Subsequently, a PHI Versa Probe III instrument was utilized to record the spectrum. The pressure of the system was smaller than $1x10^{-7}$ Torr. For excitation, a monochromatic aluminum X-ray source (Al Kα) with an energy of 1486.6 eV was used and an Ar ion beam was employed for charge removal.

Electrochemical analysis

All the electrochemical measurements were carried out on a PGSTAT128N (Autolab, Metrohm) potentiostat / galvanostat in a standard three-compartment electrochemical cell using a rotating ring-disk electrode (RRDE) with glassy carbon (GC) surface (5 mm diameter) as the working electrode (WE). A freshly prepared aqueous 0.1 M KOH solution (pH = 13) was used as the electrolyte and was purged for 30 min. prior to the experiments and through ORR measurements with *purity grade 2* N_2 or O_2 to achieve saturation. A N_2 saturated electrolyte will then be purged for at least 1 h with O_2 to achieve saturation with the other gas. A platinum wire was used as the counter electrode (CE) and a mercury oxide electrode (MOE, Hg/HgO) with aqueous 0.1 M KOH as the filling solution, was applied as the reference electrode (RE). The catalyst inks was prepared by dispersing 1.0 mg of the catalyst powder in a 250 µL mixture of distilled water, isopropanol, and 5% Nafion alcohol solution (v/v/v = 4:1:0.02) and sonicated for 30 min. prior to use. Before

drop casting the electrocatalytic ink on the electrode surface, the working electrode was polished with fine Al_2O_3 powder and thoroughly rinsed with distilled water. Afterwards, an 8.5 μL aliquot (0.034 mg & 173 μg cm^{-2} catalyst loading) of the electrocatalytic ink was drop-casted on the electrode surface and was dried under N_2 stream to furnish a thin coating film (TF-RRDE). Linear sweep voltammograms (LSV) were collected (1600 RPM, 5 mV s^{-1} scan rate) in both N_2 and O_2 saturated electrolyte at ambient temperature (24 ± 2 °C) to obtain the background-corrected LSV and outline the profile of each different catalyst.

The Koutecký-Levich (K-L) equation is the following:

$$\frac{1}{j} = \frac{1}{j_d} + \frac{1}{j_k} \quad \text{(E1)}$$

where j and j_d, and j_k are the experimentally measured, the diffusion-limited, and the kinetic current densities, respectively. The number of electrons (n) transferred in the reduction of one O_2 molecule can be determined by modifying the K–L equation as follows:

$$\frac{1}{j} = \frac{1}{B \cdot \omega^{-1/2}} + \frac{1}{j_k} \quad \text{(E2)}$$

where B (mA^{-1} cm^2 $rpm^{1/2}$) is the slope of the K-L plot and ω the rotation speed (rpm).

To construct the K-L plots, LSVs in different rotation speeds were initially recorded with the RRDE electrode. The sweep direction for LSVs was forward scan (from negative to positive potential). After normalization to the geometric surface area (A_{geom} = 0.196 cm^2), current density values on different potential values (j_{geom}) at the plateau region were noted. Subsequently, the inverse current density values (j_{geom}^{-1}) were plotted against the inverse square root of rotation speed ($\omega^{-1/2}$), and the points were fitted with a straight line. The slope of the line is equal to:

$$B = \left(0.2 \cdot n \cdot F \cdot D^{\frac{2}{3}} \cdot v^{-\frac{1}{6}} \cdot C_{O_2}\right)^{-1} \quad \text{(E3)}$$

where n is the electron transport number, F is Faraday constant (96485.332 C mol^{-1}), D is the diffusion coefficient for O_2 in water ($1.96 \cdot 10^{-5}$ cm^2 s^{-1} at temperature 25.3 °C and pressure 1 atm), *v* is the kinematic viscosity of the electrolyte (0.01 cm^2 s^{-1}), and C_{O2} is the bulk O_2 concentration in solution ($1.26 \cdot 10^{-3}$ M at pressure 1 atm). Since the O_2 bubbling was maintained throughout the entire measurement, the solution is considered O_2-saturated, thus justifying the use of the above constant.

For the calculation of electron transfer number via RRDE data (n_{RRDE}) and the H_2O_2 production yield (% H_2O_2), the following equations were used:

$$n_{RRDE} = 4 * \frac{I_D}{I_D + {}^{I_R}/_N} \quad \text{(E4)}$$

and

$$\% H2O2 = \frac{100 * 2 * \frac{I_R}{N}}{I_D + \frac{I_R}{N}} \quad \text{(E5)}$$

where I_D is the recorded current from the GC disk electrode, I_R is the recorded current from the Pt ring electrode, and N is the collection efficiency of the Pt ring, which was provided as 0.249 by the manufacturer.

Electrochemical impedance spectroscopy (EIS) was conducted from 10^5 to 10^{-1} Hz with an AC amplitude of 0.01 V. The Nyquist semicircles were constructed and the charge-transfer resistance (R_{ct}) as well as the internal/electrolyte resistance (R_s) were graphically extracted. The impedance characteristics of the materials of interest were fitted into appropriate Randles circuits (*vide infra*, **Figure S6**).

The Tafel slopes were extrapolated by plotting potential vs RHE values (V) against the kinetic current density, derived from equation E1, outlined below in equation E6:

$$j_k = \frac{(j \cdot j_d)}{j_d - j} \quad \text{(E6)}$$

The electrochemically active surface area (ECSA) was estimated through cyclic voltammetry (CV). Specifically, CVs in the range -0.8 V to 0.8 V vs MOE in N_2-saturated 0.1 M KOH electrolyte were recorded with 50 mV s^{-1} scan speed. After locating the PdO reduction peak, the charge (Q_o) was calculated via integration of the area under the peak and normalized with the charge acquired by the adsorption of oxygen monolayer on Pd surface per unit of area (Q_o^{ref} = 425 μC cm^{-2}), and the Pd loading. Mass activity (MA) is calculated by dividing the kinetic current density value with the Pd loading on the electrode. Specific activity (SA) is calculated by dividing the MA value with the ECSA value.

Chronoamperometry (CA) was employed in an O_2-saturated 0.1 M KOH electrolyte solution by recording the current density at a specific potential for 10,000 s (*ca* 3 hours).

Experimental procedures

Exfoliated semiconducting MoS_2. Bulk MoS_2 (200 mg) was mixed with 15 mL of HSO_3Cl mixture was subjected to bath ultrasonication for 8 h daily, in 30 min intervals, followed by overnight stirring (at ambient temperature), for a total of one week. Subsequently, the superacid was quenched with dropwise addition of distilled water with the mixture cooled in an ice bath, while stirring. The mixture was then filtered under vacuum through a PTFE membrane (47 mm diameter, 0.2 μm pore size) and was extensively washed until the pH of the filtrate solution is ~ 6.5-7. The collected residue was dispersed in 200 mL of NMP (~1 mg mL^{-1}) and subjected to probe ultrasonication for a total of 1.5 hrs. To maintain the sonication efficiency and rule out undesired high temperature issues, the sample was constantly kept in an ice bath. The dispersion was then transferred to a volumetric cylinder and allowed to settle overnight at room temperature. Finally, the upper 2/3 of supernatant volume were collected, filtered through PTFE membrane and

washed three times with DMF and twice with EtOH. The solid residue was dispersed in EtOH, transferred to a glass vial and the solvent was evaporated under N_2 stream. Subsequently it was dried under high vacuum to afford 40 mg of exfoliated MoS_2 as lead grey powder.

PBA stabilized Ni^0 nanoparticles 1. 1-Pyrene butyric acid (PBA, 20 mg, 0.069 mmol, 1 eq.) is dispersed in 20 mL ethylene glycol (EG) and the dispersion is combined with a 10 mL EG solution of $Ni(OAc)_2{\cdot}4H_2O$ (17.4 mg, 0.07 mmol, 1 eq.). The new dispersion is degassed with vivid nitrogen flow (N2.0, 99,9% purity) for 1 hr. Then, under continuous nitrogen flow, $NaBH_4$ (32 mg, 0.846 mmol, 12 eq.) were dissolved in 4 mL of EG containing 0.7 mL of a 1 M aqueous NaOH and the solution is quickly added into the reaction mixture. The addition is accompanied by a gradual color change from pale yellow to faint black. After 1.5 hrs of stirring at ambient r.t, the mixture is centrifuged (20 min., 6000 RPM), the pale-yellow supernatant solution was removed, and the PBA stabilized Ni nanoparticles were collected as black sediment and used in the next step without further purification.

PBA stabilized $Ni_{core}Pd_{shell}$ nanoparticles 2. The black sediment of **1** is redispersed in 10 mL EG and the mixture was degassed for 15 min. under nitrogen flow. K_2PdCl_4 (4.8 mg, 0.014 mmol) dissolved into 10 mL EG and degassed for 10 min. is slowly added into the initial mixture. After completion of the addition, the reaction mixture was left stirring vigorously for 24 hrs under nitrogen atmosphere. Afterwards, it was centrifuged (20 min., 10000 RPM) to obtain newly formed $Ni_{core}Pd_{shell}$ nanoparticles as black sediment. Upon removal of supernatant EG solution, the sediment was washed once with IPA and once with EtOH. Finally, it was collected as IPA dispersions, dried under nitrogen flow and high vacuum overnight.

PBA stabilized $Ni_{core}Pd_{shell}/MoS_2$ nanoensemble 3. In a glass vial, 6 mg of 2H-MoS_2 were dispersed in 5 mL IPA and followingly mixed with 2.5 mL of an IPA solution of $Ni_{core}Pd_{shell}$ nanoparticles (~0.6 mg mL^{-1}). The final mixture is sonicated for 4 hrs under nitrogen gas flow and left stirring 24 hrs in ambient temperature. After one centrifugation process (20 min., 6000 RPM) the supernatant is removed, and the sediment is redispersed in IPA and stored.

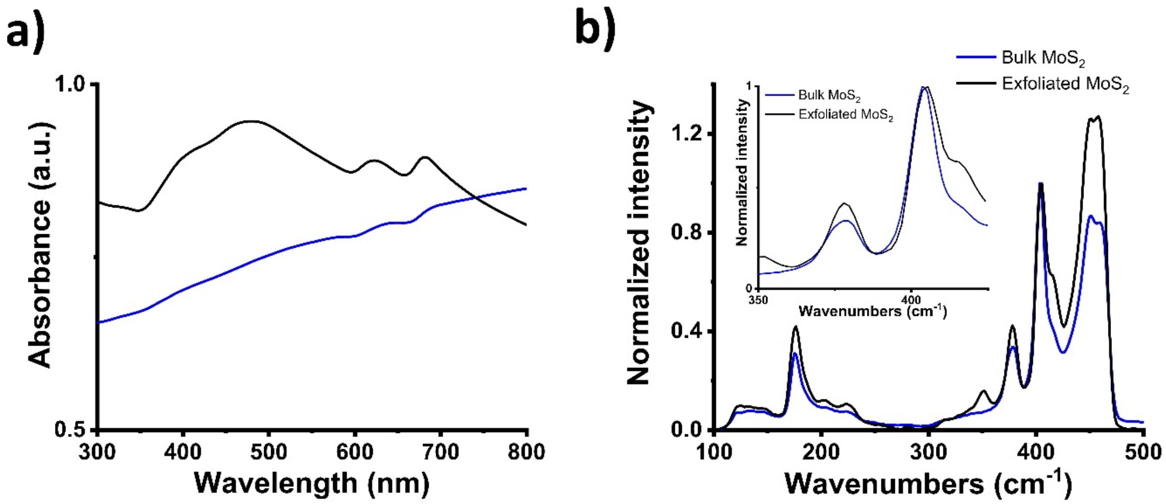

Figure S1. a) UV-vis-NIR, and b) normalized to A_{1g} mode Raman spectra (λ_{exc} = 633 nm) of bulk MoS_2 (blue) and exfoliated semiconducting MoS_2 nanosheets (black).

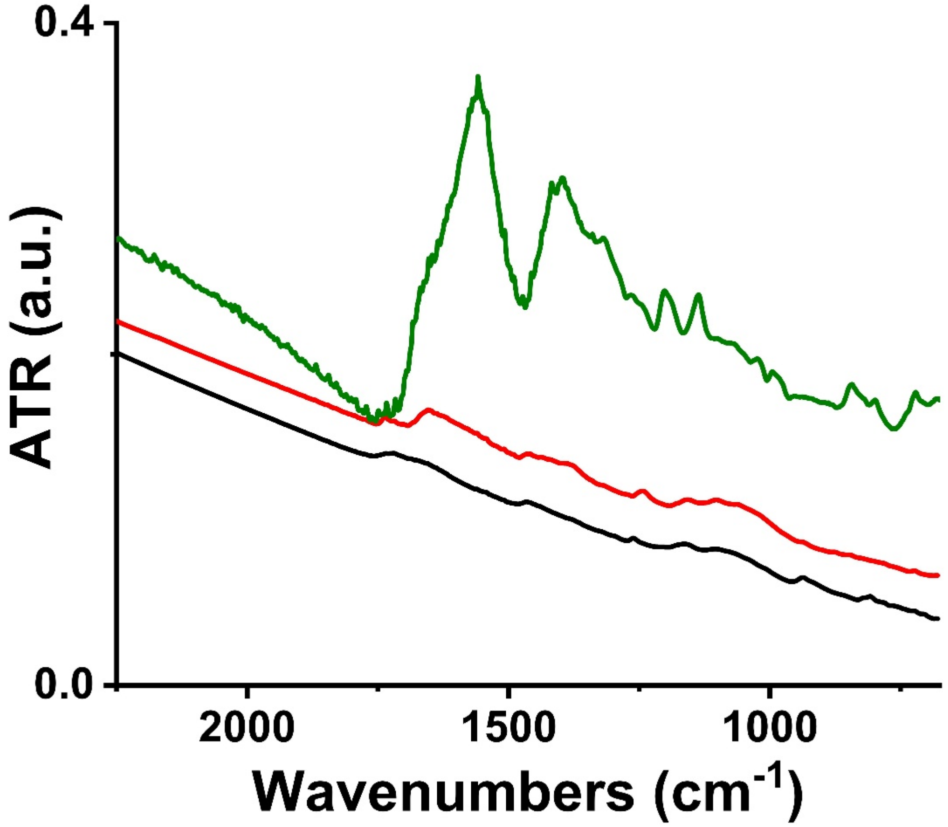

Figure S2. ATR-IR spectra on Ge crystal of **2** (olive), 2H-MoS_2 (black), and **3** (red).

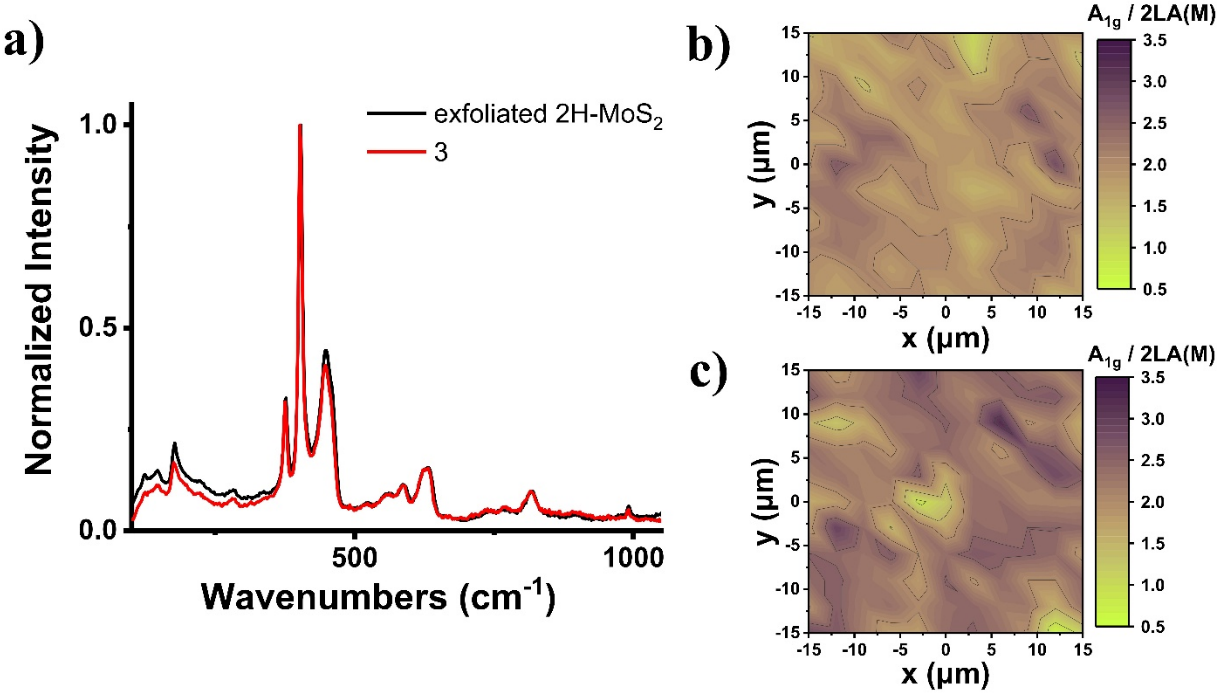

Figure S3. a) Normalized to A_{1g} mode Raman spectra (λ_{exc} = 633 nm) exfoliated semiconducting MoS_2 nanosheets (black) and nanoensemble **3** (red), b, c) Raman spectral maps of a 30 x 30 µm area of exfoliated semiconducting MoS_2 nanosheets (upper) and nanoensemble **3** (lower), respectively.

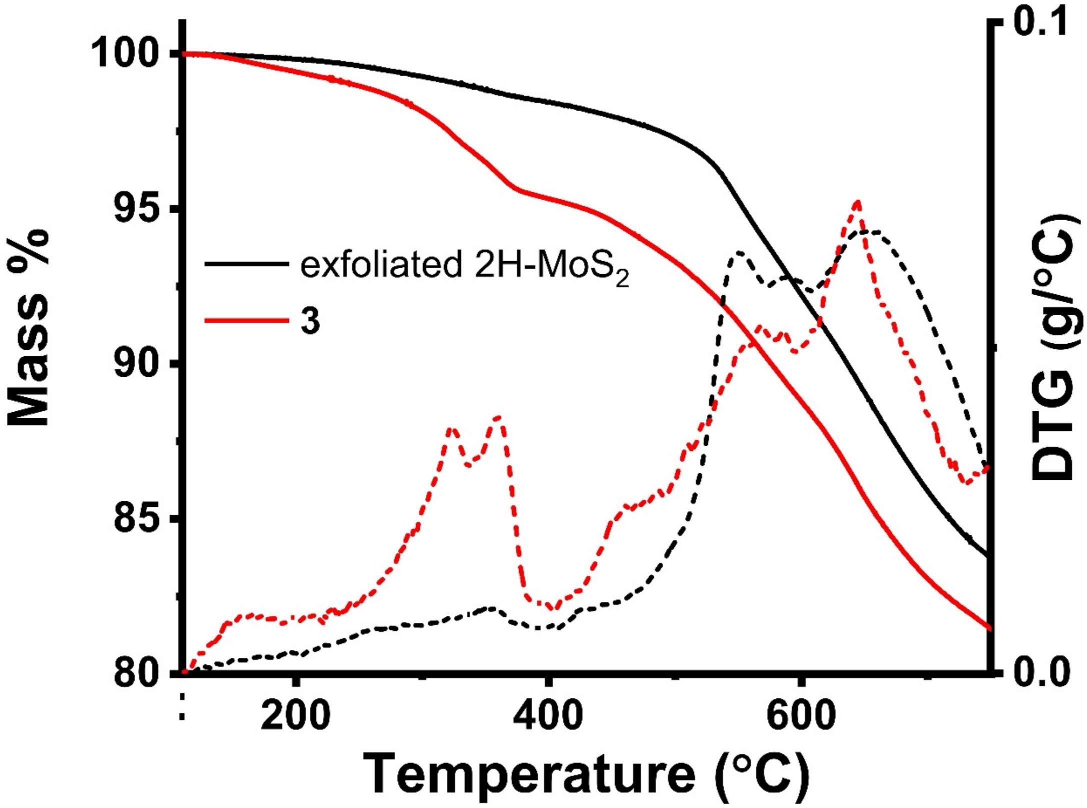

Figure S4. TGA thermographs (solid lines) and differential thermographs (dashed lines) of exfoliated semiconducting MoS_2 nanosheets (black) and nanoensemble **3** (red) under N_2 atmosphere.

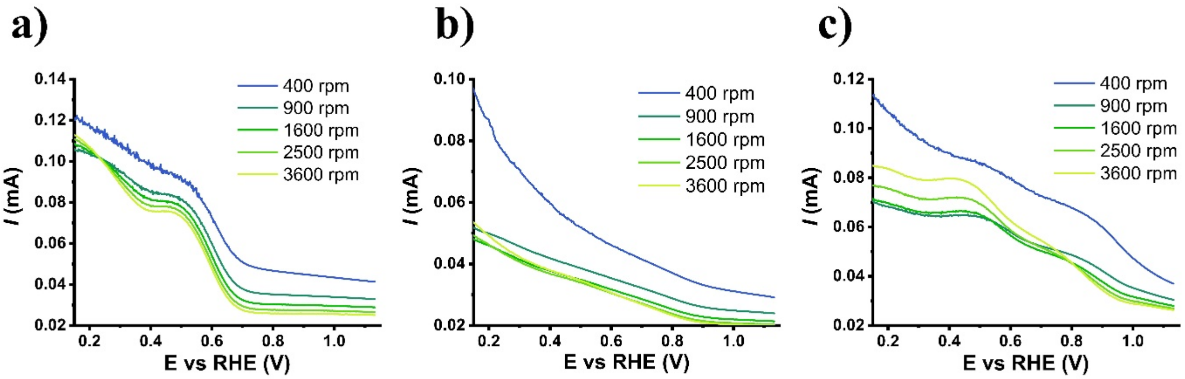

Figure S5. Ring current from RRDE response in different rotation speeds (400–3600 rpm) for a) exfoliated MoS_2, b) **3**, and c) 30 % Pd/C. All assays were performed in O_2-saturated aqueous 0.1 M KOH as electrolyte with 5 mV s^{-1} scan rate.

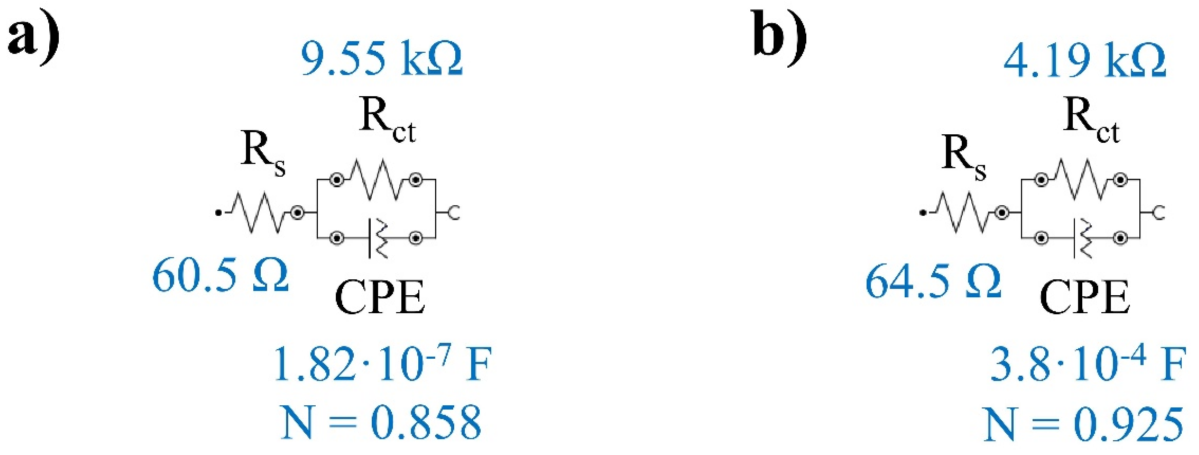

Figure S6. Equivalent circuit diagrams and respective fitted values for a) nanoensemble **3**, and b) 30% Pd/C (Randles circuits with a constant phase element, CPE).

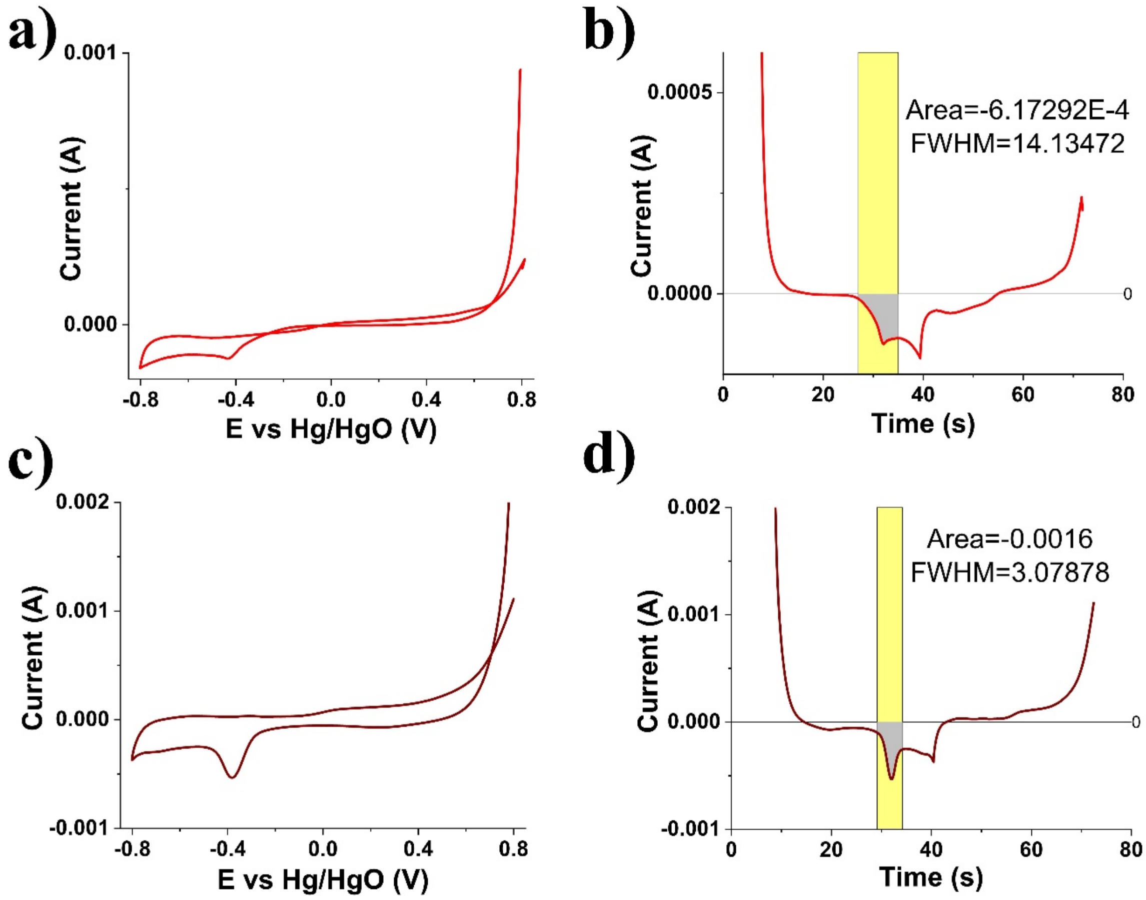

Figure S7. Cyclic voltammograms and Pd-O reduction peak charge calculation for a, b) nanoensemble **3** (red), and c, d) 30% Pd/C (wine).
